# Functional Recovery of a GCDH Variant Associated to Severe Deflavinylation—Molecular Insights into Potential Beneficial Effects of Riboflavin Supplementation in Glutaric Aciduria-Type I Patients

**DOI:** 10.3390/ijms21197063

**Published:** 2020-09-25

**Authors:** Joana V. Ribeiro, Cláudio M. Gomes, Bárbara J. Henriques

**Affiliations:** 1 Biosystems and Integrative Sciences Institute, Faculdade de Ciências, Universidade de Lisboa, 1749-016 Lisboa, Portugal; mjvribeiro@fc.ul.pt (J.V.R.); cmgomes@fc.ul.pt (C.M.G.); 2 Departamento de Química e Bioquímica, Faculdade de Ciências, Universidade de Lisboa, 1749-016 Lisboa, Portugal

**Keywords:** riboflavin, FAD, flavoenzymes, flavinylation, protein folding, spectroscopies, glutaric aciduria type I, neurometabolic disorder, inborn errors of metabolism

## Abstract

Riboflavin is the biological precursor of two important flavin cofactors—flavin adenine dinucleotide (FAD) and flavin mononucleotide (FMN)—that are critical prosthetic groups in several redox enzymes. While dietary supplementation with riboflavin is a recognized support therapy in several inborn errors of metabolism, it has yet unproven benefits in several other pathologies affecting flavoproteins. This is the case for glutaric aciduria type I (GA-I), a rare neurometabolic disorder associated with mutations in the *GCDH* gene, which encodes for glutaryl-coenzyme A (CoA) dehydrogenase (GCDH). Although there are a few reported clinical cases that have responded to riboflavin intake, there is still not enough molecular evidence supporting therapeutic recommendation. Hence, it is necessary to elucidate the molecular basis in favor of riboflavin supplementation in GA-I patients. Here, using a combination of biochemical and biophysical methodologies, we investigate the clinical variant GCDH-p.Val400Met as a model for a phenotype associated with severe deflavinylation. Through a systematic analysis, we establish that recombinant human GCDH-p.Val400Met is expressed in a nonfunctional apo form, which is mainly monomeric rather than tetrameric. However, we show that exogenous FAD is a driver for structural reorganization of the mutant enzyme with concomitant functional recovery, improved thermolability, and resistance to trypsin digestion. Overall, these results establish proof of principle for the beneficial effects of riboflavin supplementation in GA-I patients.

## 1. Introduction

Riboflavin, or vitamin B2, is the precursor of flavin mononucleotide (FMN) and flavin adenine dinucleotide (FAD), two highly important protein cofactors that participate in one- or two-electron and proton transfer reactions. These reactions are among key biochemical processes aimed at producing energy in the mitochondria as they provide electrons to the respiratory chain for ATP production [1]. Therefore, flavin availability in the cell is crucial to guarantee the function of flavoenzymes and, ultimately, adequate energy production levels [2,3]. Indeed, multiple studies have reported that decreased flavin availability due to riboflavin deficiency leads to a reduction in mitochondria β-oxidation due to diminished acyl-coenzyme A (CoA) dehydrogenase (ACDH) activity [4,5,6]. Besides, the capacity of FAD to function as a pharmacological chaperone in assisting flavoprotein folding and improving stability of the native structure has also been reported [7,8,9]. We previously investigated the effect of flavin on the conformational stability of three different ACDHs and showed that increasing amounts of exogenous flavin increased the proteins’ stability [10]. One of the ACDHs under study was glutaryl-CoA dehydrogenase (GCDH), a homotetrameric flavoprotein that localizes to the mitochondrial matrix, where it catalyzes the dehydrogenation and decarboxylation of glutaryl-CoA to yield crotonyl-CoA and carbon dioxide [11,12]. This study has shown that the presence of 2.5-fold excess of FAD is stabilizing, yielding an increase of 8 °C in the apparent melting temperature of wild-type GCDH [10]. Recently, another study using a murine tumor cell line (B16 cells) showed that cell culture in riboflavin-depleted medium leads to a decrease of 13% in the abundance of 68 flavoproteins, and one of the most affected was GCDH [13]. These reports highlight the impact of FAD availability on GCDH expression and stability and suggest that riboflavin supplementation can have an important role in the disease state.

Genetic defects in the gene that encodes GCDH cause glutaric aciduria type I (GA-I; OMIM #231670), an autosomal recessive neurometabolic disorder of tryptophan, lysine, and hydroxylysine catabolism [14]. GA-I symptoms generally include acute neurological crisis, macrocephaly, and delayed motor development. Acute encephalitic crisis can occur between 6 and 18 months of age, being usually triggered by a fever episode due to routine vaccinations or infections [14,15]. Currently, the therapy for GA-I consists of a dietary treatment with low lysine/tryptophan intake and L-carnitine and amino acid mixture supplementation [15]. As for other metabolic disorders, some patients of GA-I have been shown to benefit from riboflavin supplementation [16,17]. The first report described two GA-I patients who, upon treatment with a low protein diet and riboflavin supplementation, evidenced decreased urinary excretion of glutaric acid, one of the GA-I biomarkers [18]. Later, another study reported that both riboflavin supplementation and the administration of a γ-aminobutyric acid (GABA) analogue could prevent the progression of neurological symptoms [19]. Lipkin et al. also showed that riboflavin supplementation led to a decrease in the levels of GABA in the cerebrospinal fluid and modestly alleviated clinical symptoms [20]. Another study reported the case of a heterozygous patient for the mutations GCDH-p.Pro248Leu and GCDH-p.Ser139Leu, with high residual GCDH activity in vitro (20% of controls) [21]. Upon riboflavin supplementation, the residual activity of the enzyme increased and the urinary excretion of glutaric acid decreased, showing a true case of riboflavin responsiveness. Although these clinical cases describe a therapeutic benefit of riboflavin supplementation and some in vitro studies have shown an improvement in protein folding, this vitamin is still not recommended as a therapy to manage GA-I [22].

The aim of this work was to investigate the molecular mechanism underlying the rescue of GCDH by riboflavin supplementation and subsequently clarify its potential therapeutic effects in GA-I patients. For this, we chose the GCDH-p.Val400Met disease variant as the model, which we have previously reported to present an overall fold similar to that of wild-type, although slightly less compact, with compromised FAD interactions and a substantial residual activity in vitro [23], making it a good candidate for this investigation. We hypothesized that, as observed for other flavoproteins, improving FAD interactions would have a positive effect on enzyme folding, stability, and function. Moreover, we anticipated that these effects would probably be more relevant under adverse physiological conditions, resulting in proteome instability. To address this hypothesis, we carried out an in vitro study using biochemical and biophysical methods on the effect of FAD availability on conformation, oligomerization, stability, and proteolytic susceptibility in typical physiological conditions and under heat stress conditions, mimicking the adverse physiological factors of a fever event. The obtained results elucidate the molecular mechanism behind riboflavin supplementation in GA-I patients and suggest that other misfolded GCDH disease variants, as observed in other metabolic diseases, might also be rescued by similar mechanisms.

## 2. Results

### 2.1. Purified Human Recombinant GCDH-p.Val400Met Is Depleted of FAD Cofactor

**Figure 1:**
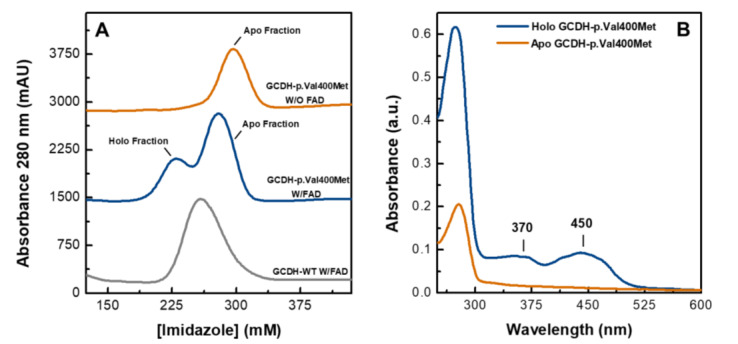
Flavin adenine dinucleotide (FAD) binding state in glutaryl-coenzyme A (CoA) dehydrogenase (GCDH)-p.Val400Met. (**A**) His-tag affinity chromatogram, bound proteins eluted with a linear gradient of imidazole from 10 to 500 mM. Elution profile of the soluble extract incubated overnight with FAD (W/FAD) in the case of wild-type (WT, gray line) and p.Val400Met (blue line) and also without the incubation step (W/O) for the variant (orange line). (**B**) UV–visible spectra of the GCDH-p.Val400Met variant, holoprotein (blue line) and apo fraction (orange line) from the His-trap column.

The human GCDH variants are expressed in *Escherichia coli* using a rich medium (details in material and methods) that is normally sufficient to guarantee that the overexpressed flavoproteins have the FAD cofactor present. Still, as reported previously, during GCDH purification, FAD is routinely added to all the buffers to ensure that purified protein has full occupancy of the cofactor site [23]. Interestingly, in the case of the GCDH-p.Val400Met variant, during purification of the His-tagged variant by affinity chromatography, the chromatogram showed two peaks between 200 and 300 mM imidazole, corresponding to two distinct bound conformers, in contrast to wild-type GCDH (GCDH-WT) that presented only one bound fraction (Figure 1A). SDS-PAGE confirmed that the two fractions corresponded to GCDH, and UV–visible absorption spectra showed that the chromatographic bands corresponded to the holoprotein form (FAD bound), which had the characteristic flavin fingerprint in visible absorption spectroscopy, and to the apoprotein form (no FAD bound) in which these features were absent (Figure 1B). This prompted us to question the importance of the FAD incubation step prior to the purification procedure and further analyze the expression and purification of this variant. Indeed, using a rich medium during protein expression but omitting the addition of FAD to the soluble extract yielded only apoprotein (Figure 1A), indicating that this variant has flawed cofactor binding. A comparison with GCDH-WT showed that omission of the FAD incubation step still yielded holoprotein. Therefore, this result suggests the possibility that in vivo, at regular FAD levels, the GCDH-p.Val400Met variant is likely not able to accomplish full cofactor binding.

### 2.2. Apo GCDH-p.Val400Met Presents a Less Compact Conformation

We then sought a detailed comparison and structural characterization of apo and holo GCDH-p.Val400Met. For this, both forms were purified to homogeneity and analyzed spectroscopically. The absence of bound FAD in the apoprotein form was further confirmed with FAD fluorescence. Although flavin emission was quenched in the holoprotein, it exhibited the characteristic emission spectrum with a maximum at 530 nm, which was not observed in apo GCDH-p.Val400Met (Supplemental Figure S1).

**Figure 2:**
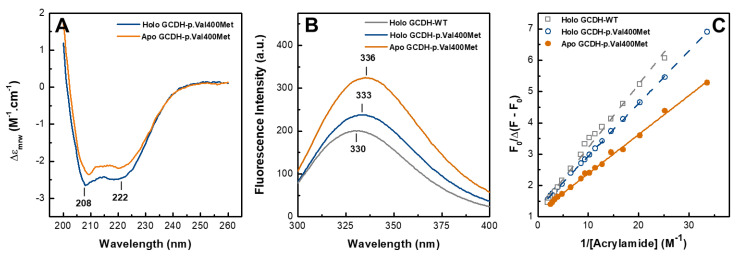
Spectroscopic analysis of holo and apo GCDH-p.Val400Met. (**A**) Far-UV circular dichroism (CD), (**B**) tryptophan emission, and (**C**) modified Stern–Volmer plot. In all panels GCDH-p.Val400Met holoprotein—blue line and open circle, GCDH-p.Val400Met apoprotein—orange line and closed circle, and WT—grey line and open squares. Protein concentration was 0.25 and 0.05 mg·mL^−1^, respectively, for far-UV CD and fluorescence emission spectra in 5 mM HEPES pH 7.8. Tryptophan emission spectra for GCDH-p.Val400Met holoprotein and WT were redrawn from [23] for comparison.

Differences in protein secondary structure were assessed using far-UV circular dichroism (CD). The spectra of both the holo- and apoprotein forms of GCDH-p.Val400Met revealed the typical α/β fold of GCDH with minima at 208 and 222 nm, respectively. Apart from a minor shift in one of the bands to 209 nm and discrete decrease in structural content not attributable to protein quantitation, there were no major alterations in the far-UV CD spectrum of the apo form versus the holoprotein, suggesting that the absence of the flavin cofactor does not affect protein folding or cause protein misfolding (Figure 2A).

GCDH has four tryptophan residues per monomer (Trp50, Trp 212, Trp225, and Trp299, numbered according to full-length GCDH), which allows using fluorescence spectroscopy to monitor changes in the conformation surrounding Trp moieties and thus infer on protein tertiary structure. Depending on solvent accessibility, Trp emission maxima can range from 310 to 350 nm, with the red-shifted emission indicating a higher solvent exposure. As shown in previous work, GCDH-p.Val400Met has a red-shifted Trp emission maximum versus that of GCDH-WT, suggesting a less compact structure. Analysis of the apoprotein revealed a more prominent 6 nm red-shift, compatible with looser tertiary contacts arising from lack of FAD binding (Figure 2B). To further assess the microenvironment surrounding Trp residues, fluorescence quenching experiments were performed. Judging from the GCDH crystal structure (PDB: 1SIQ), only two of the Trp residues seemed to be relatively solvent-exposed (Trp 50 and Trp 212, with 66 Å^2^ and 151 Å^2^ solvent accessible area, respectively). Therefore, we employed acrylamide, a neutral quencher that can interact with tryptophan residues independently of their solvent accessibility [24]. GCDH protein solutions (WT, and p.Val400Met holo and apo forms) were thus titrated with acrylamide and the emission spectra were recorded, monitoring the emission intensity at 330 nm. Data analysis using the modified Stern–Volmer plot yielded the fraction of initial fluorescence accessible to the quencher (*f_a_)* and the Stern–Volmer quenching constant (*K_a_*) (Figure 2C, Table 1). The *f_a_* value was similar for the three tested proteins and indicated that three out of the four tryptophan residues were accessible to acrylamide, in agreement with findings for other GCDH variants [25]. However, the Stern–Volmer quenching constants differed between the proteins, with the disease variant presenting higher Stern–Volmer quenching constants. This difference was more notorious when we compared the apoprotein variant with the wild-type as this variant presented a *K_a_* that was 1.5 times higher, indicating an enhancing of the quenching by acrylamide (Table 1 and Figure 2C). These results suggest that, although the same number of Trp residues is accessible to the quencher, in apo GCDH-p.Val400Met, the Trp residues are more readily accessible to the quencher, in agreement with this form presenting a less compact structure with increased dynamics.

### 2.3. Deflavinylation Affects GCDH Quaternary Structure

**Figure 3:**
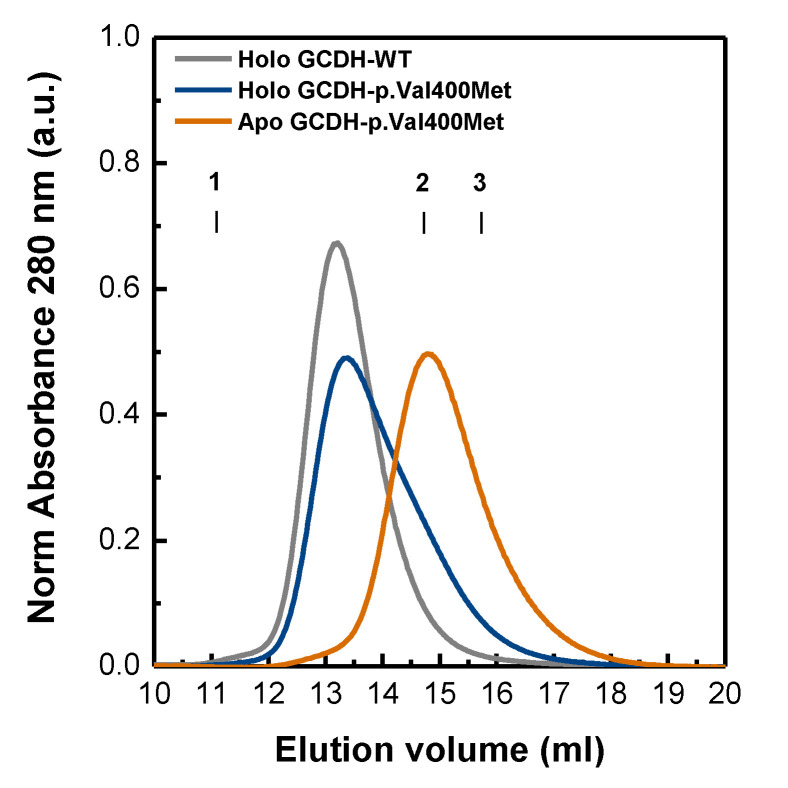
Size-exclusion chromatography of GCDH-WT and GCDH-p.Val400Met variant. Protein solutions were applied into a Superdex 200 10/300 GL column equilibrated in 10 mM HEPES pH 7.8, 150 mM NaCl. GCDH-WT—gray line, GCDH-p.Val400Met holoprotein—blue line, and GCDH-p.Val400Met apoprotein—orange line. Absorbance intensity at 280 nm was normalized in relation to total amount of protein applied. The elution volumes of ferritin (440 kDa), conalbumin (75 kDa), and ovalbumin (43 kDa) are also shown in the chromatogram as 1, 2, and 3, respectively.

We then tested whether GCDH deflavinylation would impair its quaternary assembly. Previous studies have indicated that the isoalloxazine ring of flavin is a necessary element for the ACDHs to assemble into functional tetramers that would otherwise remain as nonfunctional monomers [9]. Interestingly, the Val400Met mutation under study is located in an important region that is involved in intersubunit contacts, which implies that lack of flavin combined with the deleterious position of the mutation in the protein structure might cumulatively contribute to influence tetrameric assembly of GCDH [23,26]. Because our experiments indicated that FAD depletion altered protein compactness, we hypothesized that it could have an impact on GCDH quaternary structure. To evaluate this, we resorted to size-exclusion chromatography (SEC). The GCDH-WT elution profile showed a sharp single peak (V_e_ = 13.2 mL, ~150 kDa) corresponding to the tetrameric state of the protein (Figure 3), which was in contrast to the broader SEC profiles of the GCDH-p.Val400Met variant, suggesting a mixture of species/conformers. To interpret these results, we carried out Gaussian deconvolution analysis. We determined that the elution profile of apo GCDH p.Val400Met corresponded to single species (V_e_ = 14.7 mL, ~70 kDa), likely corresponding to a less compact monomer. On the other hand, the SEC profile of as-purified holo GCDH-p.Val400Met was best fitted to two distributions, indicating a mixture of two species corresponding mainly to a GCDH tetramer (~40%) and dimer (~60%). These results showed that the mutation and its deflavinylation impacted the quaternary structure of the protein, corroborating spectroscopic data that indicated a less compact and more dynamic fold.

### 2.4. Flavin Supplementation Rescues Apo GCDH-p.Val400Met Enzymatic Activity

**Figure 4:**
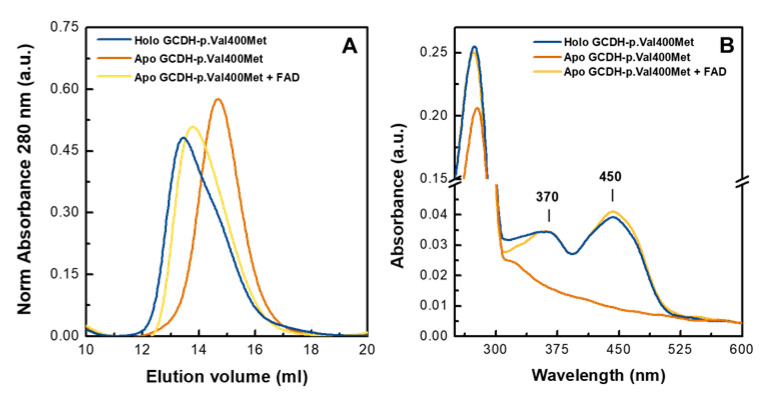
FAD supplementation rescues the folding of the GCDH-p.Val400Met clinical variant. (**A**) Size-exclusion chromatography of GCDH-p.Val400Met samples incubated overnight at 4 °C. Protein solutions were applied into a Superdex 200 10/300 GL column equilibrated in 10 mM HEPES pH 7.8, 150 mM NaCl. Absorbance intensity at 280 nm was normalized in relation to total amount of protein applied. (**B**) UV–visible absorption spectra recorded at room temperature. In both panels, GCDH-p.Val400Met holoprotein—blue line, GCDH-p.Val400Met apoprotein—orange line, and GCDH-p.Val400Met apoprotein + FAD, reconstituted—yellow line.

The data gathered in previous experiments suggests that the GCDH-p.Val400Met variant tends to be in the apo monomeric form. Accordingly, this apoprotein is nonfunctional, with no detectable residual GCDH enzymatic activity. As we observed that addition of flavin to the soluble extract during the purification process could partly rescue the holoprotein form of this variant, we investigated whether apo GCDH-p.Val400Met could incorporate the cofactor and restore catalytic competence. For this we incubated the apo GCDH-p.Val400Met variant with flavin overnight at 4 °C at a FAD/protein ratio of 2.5. This FAD supplementation was previously reported to correspond to the increase in FAD content in muscle mitochondria after riboflavin supplementation in patients with multiple acyl-CoA dehydrogenase deficiency (MADD), which also rescued electron transfer flavoprotein (ETF) deficiencies in vitro [27,28]. Control samples (holo- and apoprotein) were incubated under identical conditions in the absence of FAD. The protein samples were loaded into a size-exclusion chromatography column to evaluate its quaternary state. The profile obtained for the reconstituted protein (apoprotein incubated with FAD) showed the characteristic broad peak of this variant; however, in this reconstituted form, the maximum of the peak was shifted to an elution volume corresponding to higher molecular weight (Figure 4A). Deconvolution of the elution profile of the reconstituted apoprotein showed that it eluted mainly as a mixture of two species in a proportion of 35% and 65%, corresponding to a tetramer and dimer species (Supplemental Figure S2, Supplemental Table S1). This profile was similar to that of the holo GCDH-p.Val400Met obtained in the same condition (overnight at 4 °C) (Supplemental Figure S2, Supplemental Table S1), suggesting that reconstituted apoprotein acquires a quaternary structure similar to that of the purified holo variant. UV–visible spectra showed that the reconstituted apoprotein presented the typical visible fingerprint of the FAD cofactor (Figure 4B) with an Abs^280nm^/Abs^450nm^ = 5.7, which was identical to that obtained for the holoprotein (Abs^280nm^/Abs^450nm^ = 6.2). Considering the Abs^280nm^/Abs^450nm^ and SEC profile, we established that GCDH-p.Val400Met apoprotein in the monomeric state was able to fully incorporate the FAD cofactor, even though the GCDH tetrameric assembly was incomplete. We then determined the enzymatic activity of the reconstituted protein. Interestingly, the reconstituted protein was able to gain around 80–90% of the holoprotein activity (1683 ± 157 U·mg^−1^, *n* = 5). The difference between the enzymatic activity of the purified holoprotein and that of the reconstituted apoprotein could arise from variations in the proportion of the different species in solution as the reconstituted protein presented a slightly lower amount of GCDH tetramer. Our results show that flavin incorporation, during the purification process or in the purified apo form, partially converts (35–40%) the monomeric deflavinylated form to a catalytically competent tetrameric holoprotein, whose enzymatic activity is sufficient to maintain function above the disease threshold.

### 2.5. Flavin Improves the Conformational Quality of Holo GCDH-p.Val400Met

**Figure 5:**
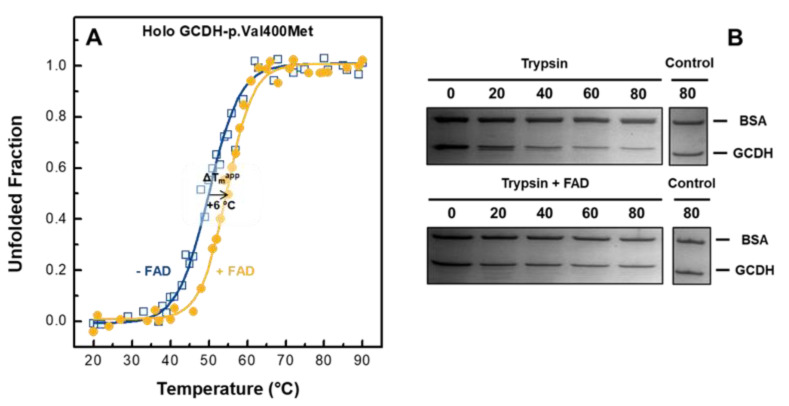
Effect of FAD on the conformational stability of holoprotein GCDH-p.Val400Met. (**A**) Representative thermal stability profiles obtained following ellipticity variation at 222 nm for the holoprotein in the absence (blue open squares) or presence (yellow closed circles) of flavin. The solid curves represent the best fits to a two-state model sigmoid from which the apparent melting temperatures were determined (*n* = 3, see Material and Methods). (**B**) Effect of FAD on the susceptibility of GCDH-p.Val400Met to proteolysis. Proteolytic digestion at 37 °C for 80 min with trypsin in 0.1 M Tris/HCl pH 8.5 in the absence and presence of 2.5-fold excess of FAD as analyzed by SDS-PAGE. Control samples were incubated under identical conditions in the absence of trypsin. Bovine serum albumin was added to the loading buffer as an internal standard and to correct for variations in the amount of loaded protein.

We already showed that high concentration of FAD could rescue apo GCDH forms to a holoprotein form that is catalytically competent. As previously reported, flavin is known to function as a pharmacological chaperone, being capable of improving protein folding and stability, such as in wild-type GCDH [10,28]. Therefore, here, we explored this capability by evaluating conformational stability changes of the p.Val400Met holoprotein in the presence of external FAD. For this purpose, we determined thermal denaturation profiles in the absence and presence of FAD, resorting to far-UV CD and fluorescence spectroscopy. The proteins were incubated overnight at 4 °C with and without a 2.5-fold excess of FAD, after which protein thermal melting was assessed, maintaining the protein/FAD ratio. The thermal denaturation profiles obtained following loss of secondary structure as monitored by CD (Figure 5A) followed an irreversible cooperative transition with an apparent midpoint unfolding temperature (T_m_^app^) of 50 °C in the absence of FAD. The presence of FAD resulted in a high stabilizing effect, yielding a dramatic increase of +6 °C on the T_m_^app^. This stabilization is similar to that reported for wild-type GCDH (∆T_m_^app^ = +8 °C) at the same protein/FAD ratio [10]. Interestingly, monitoring fluorescence tryptophan emission elicited a similar cooperative behavior and stabilizing effect, although the variation was more discrete (∆T_m_^app^ = +3 °C, Supplemental Figure S3). This smaller change can be explained by the fact that this variant presents looser tertiary structure contacts and, as reported elsewhere [23], GCDH thermal unfolding probably begins with loss of tertiary structure, which fosters cofactor dissociation followed by secondary structure destabilization, resulting in a CD-monitored higher apparent midpoint unfolding temperature.

We also investigated whether the conformational stabilization by FAD could have an impact on the proteolytic susceptibility of GCDH-p.Val400Met. Using trypsin-limited proteolysis, the propensity for degradation of the p.Val400Met holoprotein was investigated in the absence and presence of a 2.5-fold excess of FAD. This approach allows determination of the propensity for in vivo degradation of this variant as destabilized conformations with a more flexible polypeptide chain will have a higher number of trypsin cleavage sites accessible to digestion. The samples were incubated with trypsin at 37 °C for 80 min, and aliquots were drawn every 20 min for SDS-PAGE analysis (Figure 5B). Control samples without trypsin were exposed to the same treatment to exclude possible loss of protein due to precipitation effects. There was a dramatic loss of protein after 60 min of digestion (Figure 5B), corresponding to proteolytic cleavage of around 75% of the total protein (Supplemental Figure S4). The presence of FAD could revert this profile, and even after 80 min, the native conformation of the variant was preserved with only 20% of the protein being digested in the assayed conditions (Supplemental Figure S4). Taken together, these results indicate that FAD improves the protein thermal stability and induces a protease-resistant conformation, restoring the stability profile of GCDH-p.Val400Met close to that of wild-type GCDH.

### 2.6. External FAD Preserves GCDH-p.Val400Met Enzymatic Activity during Thermal Stress

**Figure 6:**
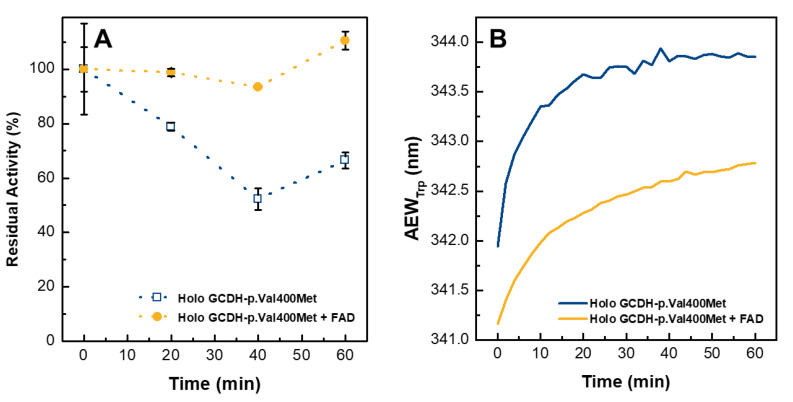
Flavin supplementation rescues activity and kinetic stability of the GCDH-p.Val400Met clinical variant during physiological thermal stress. (**A**) Kinetics of thermal inactivation of holo GCDH-p.Val400Met in the absence (blue, open squares) and presence (yellow, closed circles) of 2.5-fold excess of FAD. The proteins were incubated for up to 1 h at 39 °C, and samples were drawn every 20 min to measure the catalytic activity. The standard deviation corresponds to *n* ≥ 2 replicates. (**B**) Effect of external flavin on the loss of tertiary structure. Tryptophan emission spectra recorded every 2 min between 300 to 400 nm during 1 h at 39 °C. Average emission wavelength (AEW), which takes into consideration variation in fluorescence intensity and changes in emission maxima, was plotted as a function of time.

The acute neurological symptoms of GA-I occur, in some cases, following fever episodes linked to routine vaccinations or infections [14,15]. To investigate how the GCDH-p.Val400Met variant would behave during a fever episode and establish how dietary riboflavin supplementation would influence that behavior, we mimicked fever conditions and evaluated the loss of enzymatic activity and tertiary structure under conditions of thermal stress (Figure 6). Holoprotein samples in the absence and presence of 2.5-fold excess of FAD over protein were incubated for 1 h at 39 °C. Enzymatic activity measurements during thermal perturbation showed that enzyme function diminished 50% in 1 h, which is in agreement with previous data that indicated a loss of FAD around 40% in the same conditions [23]. Remarkably, the presence of flavin prevented this dramatic loss, and enzymatic activity remained around 90% of the initial variant activity when the reaction was carried out with FAD in solution (Figure 6A). The small observed increase in the end point in both conditions was probably due to a concentration effect associated with buffer evaporation. The assay was also performed with GCDH-WT in the absence of FAD, and in this case, the catalytic activity during 1 h at 39 °C was maintained at around 80% (data not shown), indicating that the loss of activity of the variant was due to the mutation and not because of intrinsic protein instability at the tested conditions. Moreover, it is interesting to note that FAD presence assured that the variant had activity decay similar to the one observed for GCDH-WT. Regarding the results from tryptophan fluorescence emission (Figure 6B), there was a slight increase in the average emission wavelength (AEW_Trp_) of GCDH-p.Val400Met during incubation, suggesting that the protein was becoming less compacted. However, in the presence of FAD, the initial AEW_Trp_ was slightly lower, and during incubation, it did not reach the values observed in the absence of FAD, suggesting that FAD supplementation preserved the native fold. Overall, these results indicate that if FAD levels are increased, even under physiological stress conditions, it would promote a more compact protein conformation with decreased catalytic activity loss and would therefore also likely mitigate the development of disease symptoms in patients.

## 3. Discussion

Protein misfolding induced by missense mutations lies at the root of many metabolic disorders [29]. This is no different in GA-I, a disease in which several reports have correlated functional deficiency due to missense mutations with protein misfolding and destabilization [23,25,30,31]. One strategy to rescue these misfolded proteins is resorting to the use of pharmacological chaperones, which are molecules that have the ability to stabilize the native structure and are usually ligands of the target proteins, such as cofactors, agonists, or antagonists [7,29,32]. Flavin is a well-known pharmacological chaperone, capable of promoting protein folding and stability in proteins involved in various metabolic disorders [10,28]. In patients with metabolic diseases, the increase of flavin availability in the cell is achieved through riboflavin supplementation, and although its use is recommended in some disorders [33], for GA-I, its use is not established [22,34]. To establish the proof of principle that the administration of riboflavin to patients with GA-I can, at least in some cases, be beneficial, we undertook a comprehensive study focused on the GCDH-p.Val400Met clinical variant. The choice of this specific variant was due to the fact that in a previous study, we showed that, although it presented an overall fold similar to wild-type, it had impaired FAD interactions, was slightly less compact, and exhibited lower stability [23]. Moreover, homozygous patients for the Val400Met mutation are described as having a severe phenotype and residual GCDH activity of about 10%, as measured in patient-derived fibroblasts, suggesting that, to some extent, functional tetramers exist in the cell [26]. Based on these facts, we considered that this would be a suitable model for disease phenotype associated to severe deflavinylation and therefore responsiveness to riboflavin supplementation.

Our initial analysis on the expression and purification of GCDH-p.Val400Met yielded a striking result. In contrast to wild-type and other disease variants, GCDH-p.Val400Met was expressed in two forms. One was the usual holoprotein form, and the other was the apoprotein, cofactor-free form. Moreover, we noted that lack of incubation of the soluble extract with FAD prior to purification resulted in apo GCDH-p.Val400Met, suggesting that, during *E. coli (Escherichia Coli)* expression, this variant was not able to incorporate the FAD cofactor. This result correlates with the mutation location in the GCDH structure; indeed, the affected Val residue lies in a region involving cofactor interactions [26]. As shown in our previous work using in silico methods to analyze protein structure, substitution of the buried valine for a methionine residue with higher side-chain volume will have an impact on FAD binding [23]. Therefore, it was important to first characterize the apoprotein form. Following structural analysis, we observed that flavin-depleted GCDH-p.Val400Met variant was not significantly affected in its native fold; however, we noted that the protein had a more dynamic tertiary structure as judged by combined Trp emission and acrylamide quenching assays. More dramatic was the fact that the apoprotein in solution occurred mainly in monomer state rather than the expected functional tetramer. Indeed, a previous report by Frerman et al. showed that pathogenic mutations in the carboxy terminal of GCDH led to faulty cofactor binding and impaired subunit interaction [25]. Analysis of the GCDH crystal structure revealed that the Val400Met mutation was not exactly in the C-terminal domain but rather occurred in a nearby region that involves intersubunit contacts, correlating with the results we have obtained.

**Figure 7:**
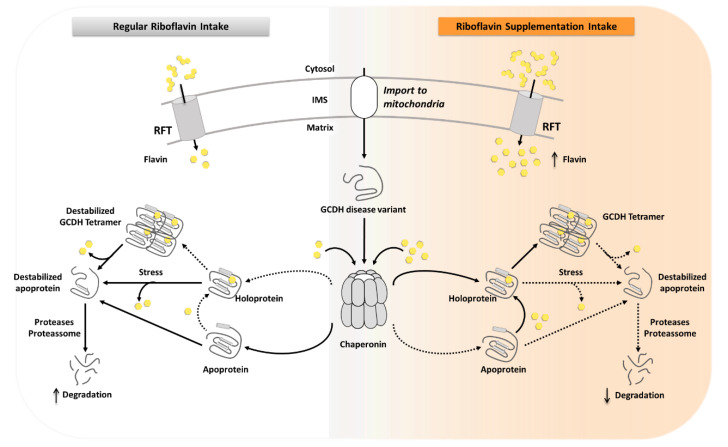
Scheme depicting the impact of FAD availability in GCDH disease variants during regular riboflavin intake versus riboflavin supplementation. After import into the mitochondria, the unfolded GCDH protein might enter the chaperonin to acquire its proper fold, as observed for other acyl-coenzyme A (CoA) dehydrogenases (ACDHs). Subsequently, the protein can follow two pathways: either the folded monomer is released from the chaperonin as an apoprotein or flavin (yellow solid hexagon) can be incorporated into the folded GCDH monomer being released as holoprotein. If the GCDH disease variant has a mutation that impairs cofactor binding, it will reduce the formation of holoprotein. Apoprotein will be conformationally unstable and targeted for degradation. In contrast, the holoprotein will assemble into a tetrameric functional structure; however, due to the disease-causing mutation, it will be prone to FAD loss and degradation. This situation will be critical under pathophysiological situations, such as fever, which will accelerate release of the incorporated FAD and increase susceptibility for degradation. Under riboflavin supplementation, flavin levels are increased in the mitochondrial matrix, resulting in an increase of GCDH holoprotein, which assembles into functional tetramers. In an environment with high FAD concentration, loss of cofactor even under stress condition is decreased, lessening the chances of degradation and improving GCDH functional capacity.

These results can explain the observed lower function of the enzyme in patient-derived cells, but an important question is whether functional deficiency could be reverted by increasing the cellular availability of FAD in patients. Riboflavin, or vitamin B2, is the precursor of FAD, whose cellular content is dependent on riboflavin transporters (*SLC52A1*, *SLC52A2,* and *SLC52A13*) and FAD synthetase [35]. During regular diet, flavin content is maintained to ensure the function of flavoproteome, and it has been reported that increasing riboflavin intake will result in an increased FAD availability [27]. To understand whether riboflavin supplementation could potentially rescue the enzymatic activity of the apo GCDH-p.Val400Met, we reconstituted this form of the protein using a 2.5-fold excess of FAD. Interestingly, the enzymatic activity of the reconstituted GCDH variant was rescued to around 80% of its holoprotein, suggesting that, in vivo, an increase in flavin availability through dietary riboflavin supplementation could be enough to rescue the inactive mitochondrial GCDH. In fact, considering that homozygous patients for the Val400Met mutation present 10% of wild-type residual enzymatic activity, we speculate that, after translation, the variant is not locked in a nonrecoverable misfolded state. Therefore, even at physiological FAD levels, some GCDH monomers are able to assemble into functional tetramers, as illustrated in Figure 7. However, as the variant presents impaired FAD binding, the assembly of native tetramers is compromised, as reported for medium-chain acyl-CoA dehydrogenase (MCAD), another member of the ACDH family. In this case, MCAD assembly into the functional tetrameric form requires flavin incorporation, or else the protein remains sequestered inside Hsp60 [9]. A similar rescue mechanism of GCDH by a chaperone, such as Hsp60, might also occur in GCDH variants (Figure 7), possibly justifying the low enzymatic activity levels measured in patient fibroblasts, in contrast with the higher levels when enzyme activity is measured in vitro for the holoprotein form [23]. This chaperone will act on GCDH, encapsulating it until FAD is incorporated, thus protecting destabilized forms from degradation.

In this scenario, FAD availability is extremely important to rescue or maintain functional forms of the GCDH-p.Val400Met variant. Our results on the effects of FAD on GCDH-p.Val400Met holoprotein showed that FAD increased protein thermal stability and induced a conformation more resistant to proteolytic digestion. Increased FAD availability can not only rescue the apoprotein forms but also stabilize and maintain the holoprotein in a more native-like conformation (Figure 7). In addition, under physiological thermal stress conditions, such as incubation at 39 °C, FAD supplementation recovered enzymatic activity and improved conformational destabilization. It should be stressed that the onset of GA-I disease symptoms, or the development of crises, are in most cases associated with infections, immunizations, and surgery during infancy or childhood that likely cause fever. Indeed, Pérez-Dueñas et al. studied two cases of homozygous patients for the GCDH-p.Val400Met variant that developed an acute encephalitic crisis due to infection (otitis and gastroenteritis) [36]. Our experiments under thermal stress, mimicking fever events, are highly suggestive of a possible role of FAD as a pharmacological chaperone. In principle, one might speculate that if a patient would be under a riboflavin supplementation regime prior to disease development, then disease onset might be mitigated as a result of stabilization of the GCDH fold and preservation of the enzymatic activity induced by flavinylation (Figure 7). It should be also known that riboflavin supplementation has no known side effects reported and is regarded as a safe nutrient supplement by regulatory agencies.

In summary, we suggest that an increase in flavin availability following dietary supplementation with riboflavin could rescue the assembly of GCDH into functional tetramers as well as mitigate protein instability resulting from physiological thermal stress events related to fever in the context of infections. Therefore, this study provides a molecular rationale of the potential benefit of riboflavin supplementation in the recovery of defective GCDH variants, which might open the possibility for the therapeutic use of riboflavin in these patients.

## 4. Materials and Methods

### 4.1. Chemicals/Materials

All reagents were of the highest purity grade commercially available. Glutaryl-CoA, FAD, 2,6-dichlorophenolindolphenol (DCPIP), phenazine methosulfate (PMS), and L-(+)-arabinose were purchased from Sigma (St. Louis, MO, USA). Acrylamide was purchased from Bio-Rad (Hercules, CA, USA).

### 4.2. Protein Purification and Biochemical Assays

*E. coli* BL21 (DE3) cells (Lucigen, Middleton, WI, USA) transformed with GCDH plasmids for the wild-type and GCDH-p.Val400Met variant were grown as described previously [23]. Briefly, cells were grown in dYT medium (16 g of Bacto tryptone, 10 g of Bacto yeast extract, and 5 g of sodium chloride) supplemented with 100 μg·mL^−1^ of ampicillin at 37 °C up to an optical density (OD) at 600 nm of around 0.5 and then induced with 0.2% of arabinose and grown overnight at 30 °C. Cells were harvested by centrifugation, resuspended in 10 mM HEPES pH 7.8, 10% ethylene glycol with 0.5 mM of phenylmethylsulfonyl fluoride (PMSF) in the presence of DNAse (PanReac, Applichem, Darmstadt, Germany), and disrupted in a French press. Routinely, soluble extracts were incubated with excess flavin at 4 °C overnight to ensure full occupancy of the FAD site before protein purification, with the exception of the GCDH-p.Val400Met apo form purification. GCDH was purified in a 5 mL HisTrap HP (GE Healthcare, Chicago, IL, USA) using a linear gradient ranging from 10 to 500 mM imidazole in 10 mM HEPES pH 7.8, 10% ethylene glycol, 300 mM NaCl, and 0.5 mM PMSF. Protein purity was confirmed by SDS-PAGE. Total protein concentration was determined using the Bradford assay, and flavin content was determined by absorption at 450 nm using the reported molar extinction coefficient ε_450 nm_ = 14,500 M^−1^·cm^−1^ [12]. GCDH catalytic activity was measured at 25 °C by monitoring DCPIP reduction at 600 nm in an assay with PMS and glutaryl-CoA [37]. One unit of catalytic activity was defined as nmol of DCPIP reduced per minute in the conditions used in the assay. For the holoproteins, the specific activity was calculated based on the total flavin content, while for the apoprotein form, it was calculated based on total protein concentration.

### 4.3. Spectroscopic Methods

UV–visible assays were performed in a SPECTROstar Nano spectrometer (BMG Labtech, Ortenberg, Germany). Far-UV CD experiments were conducted on a Jasco-1500 circular dichroism spectrophotometer (Jasco, Easton, MD, USA) using a quartz cuvette with a 1 mm path length (Hellma, Müllheim, Germany), and fluorescence studies were made on a Jasco FP-8200 spectrofluorometer (Jasco, Easton, MD, USA). CD spectrophotometer and the spectrofluorometer apparatus have a Peltier temperature control. Typically, protein concentrations were 0.25 mg·mL^−1^ (CD assays) or 0.05 mg·mL^−1^ (fluorescence). In fluorescence assays, tryptophan emission was followed using an excitation wavelength of 280 nm, and FAD emission was followed using an excitation wavelength of 450 nm; slits were set at 5 and 10 nm for excitation and emission, respectively.

### 4.4. Fluorescence Quenching with Acrylamide

Tryptophan quenching assays were performed for the holo- and apoproteins (0.2 mg·mL^−1^) using acrylamide (4 M solution), a neutral quencher. Protein solutions were titrated with acrylamide, and fluorescence spectra from 300 to 400 nm were taken using excitation wavelength at 295 nm to ensure that the light was absorbed almost entirely by tryptophan residues. Fluorescence quenching data, following intensity at 330 nm, were plotted using a modified Stern–Volmer equation:

(1) $$\frac{F_{0}}{\Delta \left(F-F_{0}\right)}=\frac{1}{f_{a}K_{a}\left[Q\right]}+\frac{1}{f_{a}}$$

where *F*_0_ is the protein’s fluorescence without the quencher, *F* is the fluorescence in the presence of quencher, *f_a_* is the fraction of initial fluorescence that is accessible to the quencher, and *K_a_* is the Stern–Volmer quenching constant of the accessible fraction [38]. Analysis of the solvent accessible area (Å^2^) of the tryptophan residues of the GCDH variant was determined by the PISA server [39].

### 4.5. Size-Exclusion Chromatography

Size-exclusion chromatography was used to assess the protein oligomeric state. Protein samples (typically 0.5 mg) were applied to a Superdex 200 10/30 column (GE Healthcare, Chicago, IL, USA), equilibrated in 10 mM HEPES pH 7.8, 150 mM NaCl, and eluted at a flow rate of 0.5 mL·min^−1^. In the flavin rescue assays, apoprotein samples (2 mg·mL^−1^) were incubated at 4 °C overnight with 2.5-fold excess of FAD. Control samples of apo- and holoprotein were also incubated at 4 °C overnight in the absence of FAD. A partial degree (10–20%) of protein precipitation was observed. Absorbance at 280 nm in the chromatographic profiles were normalized in relation to total protein applied. To estimate the distribution of the different oligomeric states of GCDH, we resorted to deconvolution of the elution profiles in two normal Gaussian distributions using OriginPro8 software (Northampton, MA, USA). The column was previously calibrated using standard proteins—ferritin (440 kDa), conalbumin (75 kDa), ovalbumin (43 kDa), and ribonuclease A (14 kDa)—and the column void volume was 9.1 mL.

### 4.6. Thermal Stability

Protein unfolding with linear temperature increase from 20 to 90 °C and a heating rate of 1 °C·min^−1^ was followed using fluorescence spectroscopy (tryptophan emission, ʎ_ex_ = 280 nm; ʎ_em_ = 330 and 350 nm) and circular dichroism (ellipticity variation at 222 nm). At least three replicates were performed for each technique. Data were analyzed according to a two-state model and fitted using CD Pal [40]. The unfolding transitions were irreversible, impeding further thermodynamic analysis, and only the apparent midpoint unfolding temperature (T_m_^app^) is reported.

### 4.7. Limited Proteolysis with Trypsin

The proteins were incubated with trypsin (bovine pancreas trypsin; Applichem) at 37 °C in 0.1 M Tris/HCl, pH 8.5, at a 10-fold excess over the protease in the absence or presence of a 2.5-fold molar excess of FAD. As a control, identical samples without trypsin were submitted to the same procedure. Protein samples were taken at different time points for 80 min, and digestion was stopped by the addition of SDS-PAGE loading buffer (3% SDS, 5% β-mercaptoethanol, and 0.1 mg·mL^−1^ of bovine serum albumin as an internal standard for the amount of loaded protein). Samples were analyzed by 12% Tris-Tricine SDS-PAGE stained with Blue Safe (NZYTech, Lisbon, Portugal), and protein quantification was done by band densitometry using a Bio-Rad Chemidoc XRS instrument (Hercules, CA, USA). The percentage of undigested protein was calculated with respect to the initial amount of protein.

### 4.8. Thermal Stress Studies

To evaluate the effect of flavin on the loss of enzymatic activity, holo GCDH-p.Val400Met (0.1 mg·mL^−1^) was incubated at 39 °C for up to 1 h in the presence of 2.5-fold excess of FAD (6.25 µM). Control samples without added FAD were also subjected to the same conditions. Every 20 min, 10 µL of the samples were collected to measure enzyme catalytic activity as described above. For the measurements using fluorescence spectroscopy, 0.04 mg·mL^−1^ of GCDH proteins were incubated at 39 °C with 150 rpm agitation in the absence or presence of 2.5-fold molar excess of flavin. The spectra were recorded following Trp emission (ʎ_ex_ = 280 nm; ʎ_em_ = 300–400 nm) at every 2 min intervals, up to 1 h.

## Figures and Tables

**Table 1:** Modified Stern–Volmer plot parameters of acrylamide quenching of tryptophan (Trp) fluorescence in GCDH.

GCDH	*K_a_* (M^−1^)	*f_a_*
Wild-Type	5.88 ± 0.42	0.84 ± 0.04
Holo p.Val400Met	7.43 ± 0.08	0.80 ± 0.01
Apo p.Val400Met	8.99 ± 0.33	0.89 ± 0.02

## Supplementary Materials

Supplementary Materials can be found at https://www.mdpi.com/1422-0067/21/19/7063/s1, Figure S1: FAD emission spectra of GCDH variants; Figure S2. SEC elution profiles of holo and apo GCDH-p.Val400Met, after incubation at 4 °C ON, deconvolution analysis; Figure S3. Effect of FAD on the conformational stability of holo-protein GCDH-p.Val400Met; Figure S4. Effect of FAD on the susceptibility of GCDH-p.Val400Met to proteolysis; Table S1. Elution profiles deconvolution analysis of holo and apo GCDH-p.Val400Met after incubation at 4 °C ON.

## Abbreviations

FMN	flavin mononucleotide
FAD	flavin adenine dinucleotide
ACDH	acyl-CoA dehydrogenase
GCDH	glutaryl-CoA dehydrogenase
GA-I	glutaric aciduria type I
GABA	γ-aminobutyric acid
CD	circular dichroism
AEW	average emission wavelength
