# Supplementary material for: Functional Recovery of a GCDH Variant Associated to Severe Deflavinylation—Molecular Insights into Potential Beneficial Effects of Riboflavin Supplementation in Glutaric Aciduria-Type I Patients
Source: Int J Mol Sci. 2020 Sep 25;21(19):7063. doi: 10.3390/ijms21197063 (PMC7583906; doi:10.3390/ijms21197063)
Supplement: Supplementary file 1 [file ijms-21-07063-s001.pdf]

International Journal of
Molecular Sciences

MDPI

Supplementary material:

Functional Recovery of a GCDH Variant Associated to Severe Deflavinylation—Molecular Insights into Potential Beneficial Effects of Riboflavin Supplementation in Glutaric Aciduria-Type I Patients

Joana V. Ribeiro [1,2], Cláudio M. Gomes [1,2] and Bárbara J. Henriques [1,2,*]

[1] Biosystems and Integrative Sciences Institute, Faculdade de Ciências, Universidade de Lisboa, 1749-016 Lisboa, Portugal; mjvribeiro@fc.ul.pt (J.V.R.); cmgomes@fc.ul.pt (C.M.G.)

[2] Departamento de Química e Bioquímica, Faculdade de Ciências, Universidade de Lisboa, 1749-016 Lisboa, Portugal

* Correspondence: bjhenriques@fc.ul.pt

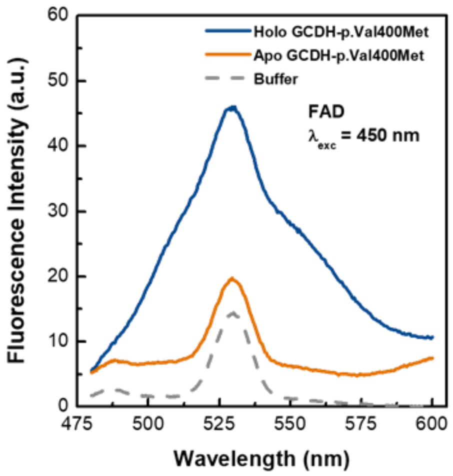

Figure S1. FAD emission spectra of GCDH variants. GCDH-p.Val400Met holo-protein, blue line, GCDH-p.Val400Met apo-protein, orange line, and buffer, grey dash line.

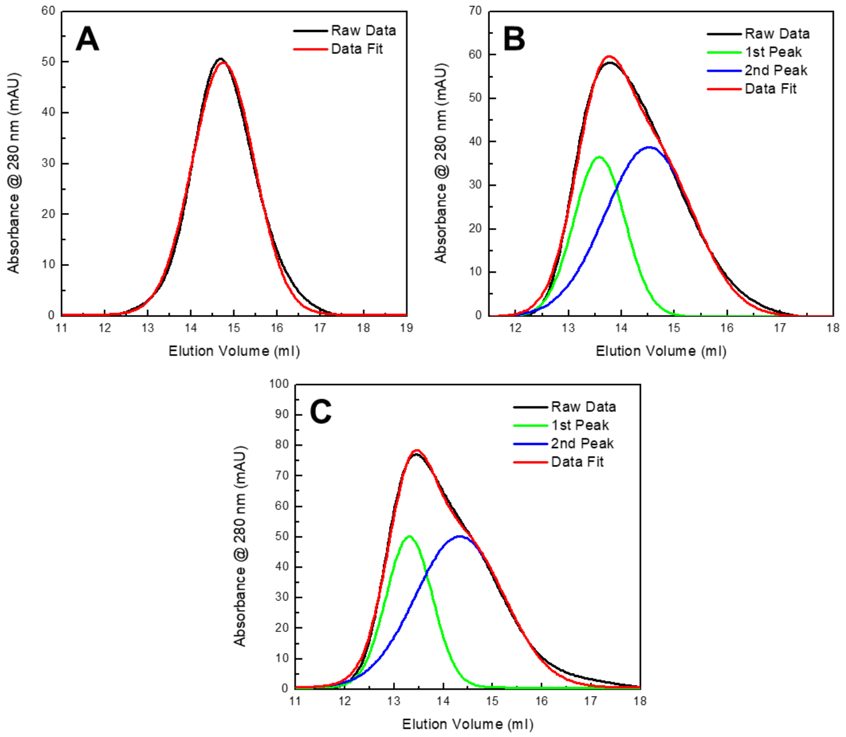

Figure S2. SEC elution profiles of holo and apo GCDH-p.Val400Met, after incubation at 4 °C ON, deconvolution analysis. To estimate the distribution of the different oligomeric states of GCDH we resorted to deconvolution of the elution profiles in one, two or three normal Gaussian distributions, here is represented the best data fit. (**A**) Apo, (**B**) apo reconstituted with FAD and (**C**) holo elution profiles and their respective data fits upon deconvolution.

Table S1. Elution profiles deconvolution analysis of holo and apo GCDH-p.Val400Met after incubation at 4 °C ON. The molecular weights corresponding to each elution volume were obtained through the calibration of the column (see material and methods). The percentage in solution of each oligomeric species was calculated using the peak area from the data obtained through deconvolution. Holo and apo reconstituted fitted two normal Gaussian distributions, while apo best fitted to a single one.

GCDH Variant	FAD	Elution Volume (mL)	Molecular Weight (kDa)	%
GCDH-p.Val400Met Holo	-	13.3	140	34
		14.3	89	66
GCDH-p.Val400Met Apo	-	14.7	74	-
	+	13.6	122	35
		14.5	81	65

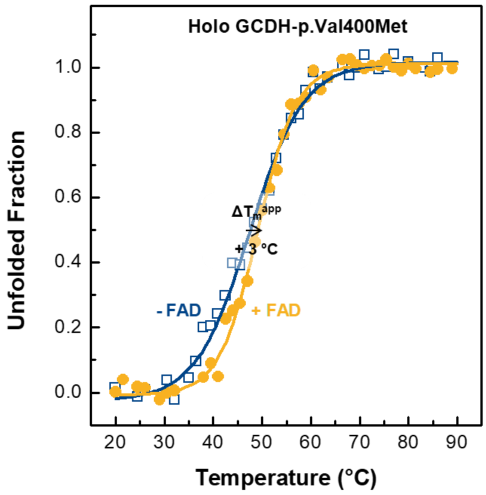

Figure S3. Effect of FAD on the conformational stability of holo-protein GCDH-p.Val400Met. Representative thermal stability profiles obtained following Trp emission for the holo-protein in absence (blue open squares) or presence (yellow closed circles) of flavin. The solid curves represent the best fits to a two-state model sigmoid from which the apparent melting temperatures were determined (n = 3, see material and methods).

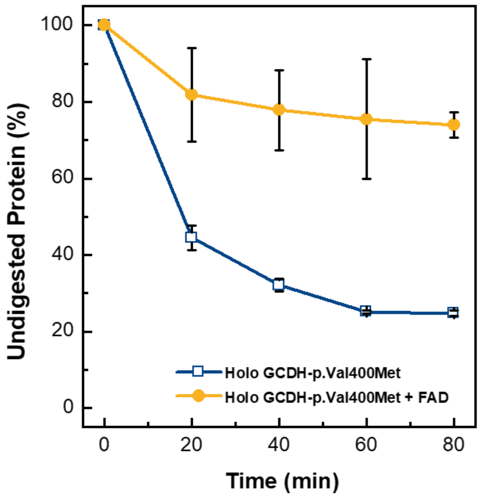

Figure S4. Effect of FAD on the susceptibility of GCDH-p.Val400Met to proteolysis. The graph corresponds to the densiometric quantitation of undigested protein. SDS-PAGE bands intensity is plotted as a percentage of undigested protein in respect to the total amount of protein in time zero. Holo-protein in absence (blue open squares) or presence (yellow closed circles) of flavin.
